# Stigmatization of obese individuals by human resource professionals: an experimental study

**DOI:** 10.1186/1471-2458-12-525

**Published:** 2012-07-16

**Authors:** Katrin E Giel, Stephan Zipfel, Manuela Alizadeh, Norbert Schäffeler, Carmen Zahn, Daniel Wessel, Friedrich W Hesse, Syra Thiel, Ansgar Thiel

**Affiliations:** 1 Department of Psychosomatic Medicine and Psychotherapy, Medical University Hospital Tübingen, Osianderstr. 5, 72076, Tübingen, Germany; 2 Institute of Sports Science, University of Tübingen, Wilhelmstr. 124, 72074, Tübingen, Germany; 3 Knowledge Media Research Center, Konrad Adenauer Str. 40, 72072, Tübingen, Germany

## Abstract

**Background:**

Weight-related stigmatization is a public health problem. It impairs the psychological well-being of obese individuals and hinders them from adopting weight-loss behaviors. We conducted an experimental study to investigate weight stigmatization in work settings using a sample of experienced human resource (HR) professionals from a real-life employment setting.

**Methods:**

In a cross-sectional, computer-based experimental study, a volunteer sample of 127 HR professionals (age: 41.1 ± 10.9 yrs., 56% female), who regularly make career decisions about other people, evaluated individuals shown in standardized photographs regarding work-related prestige and achievements. The photographed individuals differed with respect to gender, ethnicity, and Body Mass Index (BMI).

**Results:**

Participants underestimated the occupational prestige of obese individuals and overestimated it for normal-weight individuals. Obese people were more often disqualified from being hired and less often nominated for a supervisory position, while non-ethnic normal-weight individuals were favored. Stigmatization was most pronounced in obese females.

**Conclusions:**

The data suggest that HR professionals are prone to pronounced weight stigmatization, especially in women. This highlights the need for interventions targeting this stigmatization as well as stigma-management strategies for obese individuals. Weight stigmatization and its consequences needs to be a topic that is more strongly addressed in clinical obesity care.

## Background

Many obese individuals have reported perceived weight-related discrimination and stigmatization [1,2]. Stigmatization is closely linked to experiences of rejection and social exclusion, which share many characteristics with physical pain [3]. Weight stigmatization is associated with depression and low self-esteem [4] despite the widely held belief that it may motivate obese individuals to lose weight. Recent research demonstrates that it even has the opposite effect. Weight–related stigma reduces the probability of overweight individuals adopting weight-loss behaviors [5] and amplifies the link between central adiposity and non-diabetic glycemic control [6]. In a recent experimental study, obese women who were exposed to weight stigmatizing material in a film consumed significantly more calories than obese women who watched a neutral video [7]. Weight- related stigmatization, hence, contributes to the maintenance of overweight and is one factor perpetuating the vicious circle of obesity [8]. Weight-related stigmatization is therefore recognized as a public health problem [5]. This makes weight discrimination and stigmatization highly relevant for health-care professionals working with obese individuals.

Weight stigmatization has been reported in work settings, affecting nearly every area of employment including labor market access, job placement, promotion and wages [9]. For instance, there is a large body of evidence from laboratory studies on hiring decisions, which shows that fictional obese job applicants are less likely to be chosen for employment than normal-weight applicants with identical qualifications [9]. This weight stigma affects women more strongly than men [9,10]. However, current evidence mostly comes from self-report and laboratory studies. The predominant reliance on student samples in these laboratory studies, where students are asked, e. g., about hiring decisions, limits the ecological validity of the experimental evidence [11]. In contrast, human resource (HR) professionals are trained and experienced in employee appraisal, have a de facto impact on employees’ work lives and should be aware both of discrimination issues and common judgmental errors. While the few studies investigating HR professionals report weight stigmatization [12-16], most of them date back to the 1990s and are limited to sales positions. Gender [17] and ethnicity [18] are two other potential sources of stigmatization in work settings that have long been recognized and targeted by political and legal initiatives. For instance, the German Government recently announced imposing a gender quota in management positions to create equal opportunities for women in the workplace. Recent survey data from the USA has demonstrated that the prevalence of reported weight discrimination in employment settings was comparable, or in some cases, higher than reported rates of gender and race discrimination, especially in women [1].

Research on HR professionals’ beliefs about and behaviors towards obese employees is highly important in evaluating the validity of current weight stigmatization evidence. In order to validate earlier findings from surveys and laboratory studies and to provide updated evidence on a potential weight bias in HR professionals, we conducted an experimental study using a sample from a real-life employment setting. Using a computer-based paradigm, we asked HR professionals from a broad range of industries and employers to evaluate standardized photographs of individuals differing in gender, ethnicity, and Body Mass Index (BMI) in relation to employment access, work-related prestige and career achievement. By integrating gender and ethnicity, we not only accounted for the two main other sources of stigmatization in work settings, but also avoided effects of salience. If weight had been the sole prominent characteristic used to differentiate the photographs, a higher weight would have been specifically salient compared with the other stimuli presented. We also assessed processing time for each task to estimate attitude accessibility and social desirability, as HR professionals are specifically trained to take discrimination issues into account. Attitude accessibility concerns the speed with which an attitude is activated from memory. It is considered an indicator of the strength, stability and predictive value of an attitude or behavior. Computer assisted survey research has demonstrated that easily accessible attitudes are associated with shorter response times compared to less accessible attitudes. Situative or personal factors interfering with task completion, such as social desirability, slow down response times [19].

Based on previous research, we hypothesized that a) HR professionals would evaluate obese individuals lowest and normal-weight individuals highest on work-related prestige and achievement, b) obese individuals would be the least likely to be hired by HR professionals and c) that this potential weight bias would be more pronounced for obese women than men.

## Methods

### Inclusion criteria for participants

To be eligible to participate in the study, individuals had to a) be currently working in HR and b) regularly make career decisions such as assessment or employment about employees.

### Stimuli

We prepared 12 portrait photographs to be used in a computer-based experimental paradigm. The photographed individuals were aged 40 to 50 and had a higher education. To avoid effects of salience, we included gender and ethnicity as two other dimensions besides body weight into the stimulus material. Gender distribution was equal. Two male and two female individuals were obese (average BMI of 37.9 kg/m²) and the other individuals were normal-weight (average BMI of 22.4 kg/m²). Of the normal-weight individuals, two males and two females had immigrant backgrounds and were identifiable as ethnic minorities.

All individuals were photographed frontally displaying the face and the upper torso, and wore a white T-shirt. This was to standardize the photographs as much as possible.

### Experimental paradigm

We developed a computer-based experimental paradigm that randomly presented the standardized stimuli and assessed processing times (http://www.personalunitue.de/index.php?show=init&rec=y). For validation purposes, we conducted a pilot study with 30 HR professionals recruited from different companies.

Depending on the task, photographs were either presented separately or six simultaneously on a computer screen (see next paragraph on tasks and measures). The computer program randomized photograph selection and display position. Lastly, the paradigm assessed the study participant’s socio-demographic information.

### Tasks and measures

We designed three tasks that were intended to cover a broad range of work-related issues since work place stigmatization has been reported to affect nearly every area of employment [9]. These tasks included judgments related to employment access, work-related prestige and career achievement.

a) *Allocating one out of six professions to a photographed individual*: Study participants were asked to allocate one out of six predefined professions to the presented individual. The exact instructions read as follows: “Please allocate the following presented individuals to one of the designated professions. It is possible to allot multiple individuals to the same profession.” Photographs were presented separately. We predefined two professions as high prestige (medical doctor and architect), two as medium prestige (optician and retailer) and two as low prestige (usher and cleaner) according to the Standard International Occupational Prestige Scale (SIOPS) [20].

b) *Disqualifying one out of six individuals from being hired*: Study participants were asked to disqualify one out of six displayed individuals. The exact instructions read as follows: “Very often, the first impression a hiring manager receives from a job application photo influences his or her choice of job applicant. Which of the following presented individuals would you by no means hire? Please indicate your choice by clicking on the respective photograph.” Six photographs were presented simultaneously, displaying a non-ethnic normal-weight female and male, an ethnic normal-weight female and male and an obese female and male.

c) *Nominating three out of six job candidates to a supervisory position*: Study participants were asked to nominate three out of six displayed equally qualified individuals they considered to be short listed for a supervisor position. The exact instruction read as follows: “The following presented individuals applied for a supervisory position within a company in March 2008. Generally, all applicants were well suited for the job. Which three applicants do you think were short listed for the position? Please indicate your choice by clicking on the respective photograph.” Six photographs were presented simultaneously, displaying a non-ethnic normal-weight female and male, an ethnic normal-weight female and male and an obese female and male. The fictional supervisor position represented high occupational achievement and prestige.

We defined stigmatization using two different approaches: The first approach is based on the idea that in the absence of stigmatization, there should be an equipartition of occupational achievement and prestige. Equipartition means an equal distribution of achievement and prestige across individuals, irrespective, e.g., of gender, ethnicity and BMI. Therefore, we considered the divergence from equipartition in the observed allocation and nomination data as a stigmatization tendency. However, as this perspective is based on an assumptive unbiased society, we wanted to ensure that we would not classify HR professionals’ evaluations as biased when they reproduced actual ratios within society. Therefore, we applied the second approach, which is based on the idea that in the absence of stigmatization, occupational achievement and prestige should be distributed as it is within German society when taking into account gender, ethnicity, and BMI. Here, we considered the divergence in the observed allocation and nomination data from the actual data within German society as a stigmatization tendency. In this approach, we reverted to representative data obtained from the German Federal Health Survey 1998–1999 [21,22] on socio-economic strata, gender, BMI, and immigration backgrounds.

The computer assessed processing times for each task as the time between presenting the task on the screen and when the study participant completed the task.

### Procedure

A research assistant approached potential study participants in the foyer of a human resources trade show in Cologne, Germany, asking passersby if they would like to participate in a scientific study dealing with the perception and assessment of other individuals. Hence, participants were blind to the study’s specific nature and hypotheses. If the respective individual was interested in participating in the study, the research assistant asked them about their profession and specific job responsibilities in order to evaluate the inclusion criteria. If inclusion criteria were fulfilled and the individual agreed to participate, the participant was transferred to a table equipped with a laptop in a quiet area of the foyer. The research assistant gave brief introductions into how to use the laptop and started the computer-based experimental paradigm. The computer-based paradigm gave all needed instructions and guided participants through the different tasks. They consecutively worked on the three previously described tasks. Lastly, the participants were asked for their socio-demographic information.

### Ethics approval

The University of Tübingen medical faculty ethics committee approved the study (241/2011BO2). We conducted the study in accordance with the Helsinki Declaration of 1975.

### Statistical analysis

All data was analyzed using PASW Statistics 18.

To analyze for possible stigmatization tendencies concerning profession allocation, we used the Χ²- test to compare the observed allocations with the equipartition and representative data on socio-economic strata, gender, BMI and immigration background from the German Federal Health Survey 1998–1999 [21,22]. We used this survey’s data because it is the most recent data available for Germany, which reports on both socio-economic strata and BMI. For this survey, the BMI was not self-reported, but measured.

Using the Χ²- test, we compared the observed disqualifications and selections with equipartition to analyze for possible stigmatization tendencies concerning the disqualifying of individuals from being hired and selecting job candidates for a supervisory position. We calculated residuals to indicate any post-hoc differences. To determine any possible differences in stigmatization tendencies between female and male HR professionals, we conducted a secondary Χ²- test using 2 × 2 cross tables to examine profession allocation choices and the disqualification and selection of photographed individuals against the gender of the study participants.

We calculated odds ratios for the category of selecting job candidates for a supervisory position to measure the equality of opportunity. This allowed us to quantify and compare weight, gender, and race biases in selection behavior and to estimate the separate influence of weight and gender on selecting job candidates.

To analyze attitude accessibility and social desirability, we used one-way analysis of variance to assess possible differences in processing times.

## Results

### Participants

127 HR professionals meeting the inclusion criteria participated in the study. Table 1 displays the characteristics of the study participants. The largest participant subgroup worked in trade and industry (23.4%), followed by health and welfare (16.4%). Companies of all sizes were equally represented.

**Table 1:** Characteristics of study participants

**N**	**127**
Women, No. (%)	56 (44.1)
Age, mean (SD), y	41.1 (10.9)
BMI^a^, mean (SD), kg/m²	25.0 (3.4)
German nationality, No. (%)	106 (95.5)
Matriculation standard, No. (%)	108 (85.1)
Work experience, mean (SD), y	17.3 (11.1)

### Allocation of professions

As Table 2 displays, the distribution of occupational prestige, as allocated by HR professionals, differed significantly for all six individuals, both from the distribution of occupational prestige within German society and equipartition. An inspection of the residuals revealed that obese men and women were allocated significantly less often than expected to professions of high and medium prestige and significantly more often to professions of low prestige. In contrast, normal-weight individuals were allocated significantly more often than expected to professions of high prestige based on the distribution of occupational prestige within German society. While the distribution of low prestige professions within German society was correctly estimated for non-ethnic normal-weight individuals, both ethnic normal-weight individuals were allocated significantly less often to low prestige professions than expected.

**Table 2:** Allocation of professions with high, medium and low occupational prestige to six photographs by human resource professionals

**Occupational prestige**	**Observed N (%)**	**Expected N (%) in German Society**^**a**^	**Residual**^**b**^	***P *Value**	**Expected N (%) in an Unbiased Society**	**Residual**^**b**^	***P *Value**
	***Obese Women (n = 127)***						
High	3 (2.4)	20.8 (16.3)	−17.8		42.3 (33.3)	−39.3	
Medium	24 (18.9)	41.6 (32.8)	−17.6	<.001	42.3 (33.3)	−18.3	<.001
Low	100 (78.7)	64.5 (50.8)	35.5		42.3 (33.3)	57.7	
	***Obese Men (n = 126)***						
High	7 (5.5)	35.4 (28.1)	−28.4		42 (33.3)	−35	
Medium	32 (25.4)	42 (33.3)	−10	<.001	42 (33.3)	−10	<.001
Low	87 (69.1)	48.6 (38.6)	38.4		42 (33.3)	45	
	***Non-ethnic Normal-weight Women (n = 124)***						
High	54 (43.6)	20 (26.3)	34		41.3 (33.3)	12.7	
Medium	38 (30.6)	71.4 (57.4)	−33.4	<.001	41.3 (33.3)	−3.3	.044
Low	32 (25.8)	32.6 (16.4)	−.6		41.3 (33.3)	−9.3	
	***Non-ethnic Normal-weight Men (n = 126)***						
High	64 (50.8)	41.0 (32.5)	23.1		42 (33.3)	22	
Medium	43 (34.1)	66.7 (52.9)	−23.7	<.001	42 (33.3)	1	<.001
Low	19 (15.1)	18.4 (14.6)	.6		42 (33.3)	−23	
	***Ethnic Normal-weight Women (n = 127)***						
High	26 (20.4)	19.1 (15.5)	7		42.3 (33.3)	−16.3	
Medium	69 (54.3)	57.2 (44.8)	11.9	.003	42.3 (33.3)	26.7	<.001
Low	32 (25.2)	50.8 (39.8)	−18.8		42.3 (33.3)	−10.3	
	***Ethnic Normal-weight Men (n = 126)***						
High	57 (45.2)	18.9 (15.5)	18.9		42 (33.3)	15	
Medium	53 (42.2)	56.7 (44.8)	−3.7	<.001	42 (33.3)	11	<.001
Low	16 (12.6)	50.4 (39.8)	−34.4		42 (33.3)	−26	

^a^according to data from the representative German Federal Health Survey 1998/1999.

^b^values ≥ ± 2.0 correspond to p ≤ .05, values ≥ ± 2.3 correspond to p ≤ .01.

Female and male HR professionals did not differ in their allocation of the six displayed individuals (*p* > .05) to the predetermined professions.

Average processing time for profession allocation was 12.8 ± 1.7 s. There was no difference in processing times between profession allocation to the different groups of individuals (*F*(2)] = 1.852; *p* > .05).

### Disqualifying an individual from being hired

**Figure 1:**
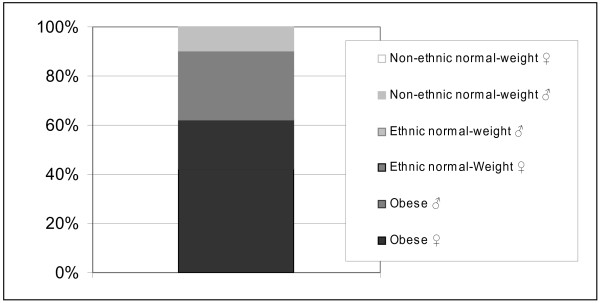
** Percentage of disqualification to be hired by HR professionals.** Legend: Segments display the percental proportion of HR professionals indicating that they would hire the respective individual by no means. HR professionals were asked to disqualify one out of six individuals. Expected percentage of disqualification for each individual on the grounds of equipartition is 16.6%. Segments are displayed in ascending order from bottom to top with respect to magnitude of portion.

42% of HR professionals disqualified the obese female when asked whom of the six displayed individuals they would absolutely not hire. Figure 1 shows that the obese female was most often and the non-ethnic normal-weight female was least often disqualified from hiring consideration. The observed proportion of hiring disqualification differed significantly from equipartition (*Χ² (N =* 127*)* = 73.727, *df* = 5, *p* < .01). An inspection of the residuals revealed that both obese individuals and the ethnic normal-weight female were significantly more often disqualified than equipartition predicted (*p* < .01). The ethnic normal-weight male and both non-ethnic normal-weight individuals were significantly less often disqualified than expected by equipartition (*p* < .01).

Female and male HR professionals did not differ in disqualification behavior (*Χ² (N =* 127*)* = 6.979, *df* = 5, *p* > .05).

The average processing time to disqualify an individual from hiring consideration was 22.7 ± 13.0 s. There was no difference in processing times between the different photographed individuals (*F*(5) = 0.985; *p* > .05).

### Selecting candidates for a supervisory position

**Figure 2:**
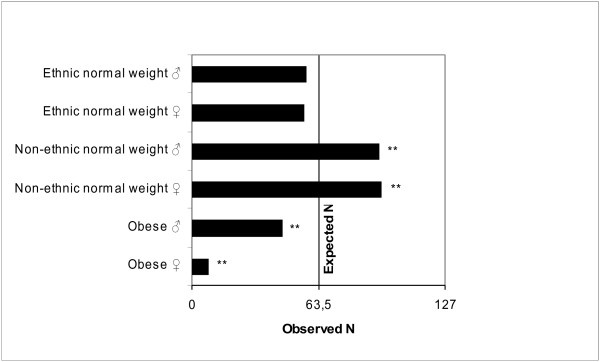
** Nomination of candidates to a fictional supervisor position by HR professionals.** Legend: Bars display the frequency with which HR professionals nominated the respective individual for a supervisor position. HR professionals were asked to nominate three out of six individuals. Expected frequency of nomination for each individual on the grounds of equipartition is 63.5%.

As Figure 2 displays, the selection frequency of obese and non-ethnic normal-weight candidates to a supervisory position differed significantly from equipartition, with the obese candidates nominated significantly less often and the non-ethnic normal-weight candidates nominated significantly more often. The selection frequency of qualified ethnic normal-weight candidates to a supervisory position did not significantly differ from equipartition.

There were significant associations between selection for a supervisory position and weight, gender, and ethnicity, with weight producing the largest inequalities in selection (see Table 3): While gender and race biases ranged from .47 and .59 in favor of male and non-ethnic candidates, respectively, n, normal-weight candidates had a 4.6-fold higher chance of being nominated compared with obese candidates. If weight was held constant, the gender bias disappeared with normal-weight females and males having equal chances of being nominated (see Table 4). However, if both the male and female candidate were obese, the gender bias reoccurred with the obese male having a 7.3-fold higher chance of being nominated compared with the obese female. If gender was held constant, a weight bias emerged, which by far more was pronounced in females. Normal-weight males had a 4.6-fold and normal-weight females had a 49.0-fold higher chance of being nominated compared with the obese female.

**Table 3:** Odds ratios for the nomination for a supervisor position by HR professionals depending on weight, gender and ethnicity

**Weight Bias**		**Gender Bias**		**Race Bias**	
Obese versus Normal-weight Individuals		Females versus Males		Ethnic versus Non-ethnic Individuals	
OR (95% CI)	%	OR (95% CI)	%	OR (95% CI)	%
0.18	455	0.68	47	0.63	59

OR = Odds Ratio, CI = Confidence Interval.

**Table 4:** Odds ratios for the nomination for a supervisor position by HR professionals depending on interactions of weight and gender

**Obese Females versus Normal-weight Females**		**Obese Males versus Normal-weight Males**		**Obese Female versus Normal weight Male**		**Obese Male versus Normal-weight Female**		**Obese Female versus Obese Male**		**Normal-weight Females versus Normal-Weight Males**	
OR (95% CI)	%	OR (95% CI)	%	OR (95% CI)	%	OR (95% CI)	%	OR (95% CI)	%	OR (95% CI)	%
0.02	4900	0.18	456	0.02	4900	0.19	426	0.12	733	1	0

OR = Odds Ratio, CI = Confidence Interval.

Female and male HR professionals showed a comparable selection behavior for both obese individuals, both non-ethnic normal-weight individuals and the ethnic normal-weight male. There was a significant gender difference for the ethnic normal-weight female though, with female HR professionals selecting her for a supervisory position significantly more often than male HR professionals (*Χ² (N =* 127*)* = 5.154, *df* = 1, *p* < .05).

## Discussion

In an experimental study using a computer-based paradigm, we asked HR professionals to judge individuals of differing gender, ethnicity, and BMI displayed in standardized photographs in regard to hiring, work-related prestige and achievement.

Overall, HR professionals showed an overestimation of occupational prestige in normal-weight individuals and an underestimation in obese individuals. For the latter group, this was true based both on equipartition and the actual distribution of occupational prestige within German society. Only 2% of study participants credited the obese women as having a high-prestige occupation such as a medical doctor or architect. When asked whom they absolutely would not hire, HR professionals showed a strong weight stigmatization. 42% disqualified the obese female and 19% the obese male. Similarly, they favored non-ethnic normal-weight candidates for a supervisory position, while rarely selecting obese individuals. Only 6% of study participants considered the obese female suitable to be a supervisor. While there was also an overall mild gender and race bias in supervisor position selections, weight produced by far the largest inequalities, especially in women. To the contrary, HR professionals showed no gender bias for normal-weight candidates, selecting females and males in equal proportion for a supervisory position. In general, we found no gender differences in stigmatization tendencies, indicating that both female and male HR professionals share similar attitudes towards obese individuals.

We interpret our study’s data as strong evidence of stigmatization against obese individuals by HR professionals. Such stigmatization affects a broad range of work-related aspects including labor market access and advancement possibilities. This weight- related stigmatization was most pronounced for obese women. These results are in line with our hypotheses and validate the self-report data and results of prior laboratory studies on weight stigmatization in work settings. Interestingly, HR professionals considered normal-weight females and males equally suitable for a supervisory position, although in Germany, there is still a significant real-life gender difference in management with only 33% of positions held by females [23]. This suggests that HR professionals are sensitized to the gender bias in work-settings as they even compensated for real-life circumstances by being more fair in gender selection than expected by gender distribution in work-life. In contrast, the HR professionals seemed to be hardly aware of a weight bias.

Our results suggest that obese individuals, especially women, are at a significant disadvantage for occupational advancement and prestige. As other research in this field demonstrates, weight stigmatization has serious health consequences as well as socio-economic and psychosocial implications. In a recent review, Puhl and Heuer [5] demonstrated that weight stigmatization not only impairs the health of obese individuals, but also leads to health disparities and, most markedly, impairs effective obesity treatment and contributes to continued excessive weight. The obesity stigma, therefore, is a priority for public health and obesity care [7]. Data from nationwide representative surveys on labor market participation supports this view, as obese workforce, especially women, are underrepresented [24-26]. Additionally, not being hired or promoted to a supervisory position can often result in an income disadvantage. There is strong evidence from large surveys on such an income disadvantage for obese women [27,28], while evidence for men is still inconclusive. Individuals who report stigmatization experiences due to their weight have lower self-acceptance and self-esteem and are more often depressed [4,29]. In the case that obese individuals manage to become hired or promoted to supervisors, there is a high probability that they will nevertheless encounter stigmatization as these tendencies are widespread in work-life, as our data shows. This can also result in the previously mentioned mental health consequences.

Our data strongly suggest that interventions targeting this type of stigmatization tendency in HR professionals should be a high priority due to its significant and complex impact on the individual and society. Concepts favoring differentiated knowledge about obesity and working against stigmatization must be developed to better educate HR professionals. Campaigns in the fields of gender and race biases are encouraging examples demonstrating that retraining thinking on this topic is possible. In clinical obesity care, weight stigma and possible stigma management strategies should also be topics discussed with patients since other obesity-related aspects such as stigmatization experiences impair effective obesity treatment.

One of our study’s major strengths is its incorporation of HR professionals from a broad range of industries and employers. The investigated sample represented a group of qualified experts with a mean professional experience of 17 years in HR. The majority of earlier studies on work stigmatization have relied on self-report and samples of lay people. Our data, thus, provides insight into the attitudes and behaviors of those people who actually make staff hiring decisions. Further strengths include the use of an objective computer-based paradigm, standardized stimulus material, processing time assessment and the results comparison with representative nationwide data.

Limitations are that we do not have data on HR professionals who were reluctant to participate in this study and that we did not include résumés of the respective individuals and HR professionals. Thus, participants had to base their decision solely on the applicant’s picture. Other limitations are that we did not include ethnic obese individuals into the stimulus material and we did not collect information on the study participants’ perception of the photographed individuals, e.g., with respect to weight or attractiveness. Additionally, our study’s design does not allow for conclusions concerning the underlying mechanisms of stigmatization.

More field and clinical studies are needed, which include samples from real-life employment settings investigating weight stigmatization in work-life. Another possible research perspective includes a more systematic investigation of other areas of life where obese individuals encounter stigmatization. There is evidence that weight stigma is also a problem in healthcare [30]. However, as with the employment field, there is still mostly only self-report data available while field studies are lacking. Studies on weight stigma should also target possible mechanisms of this phenomenon since this evidence would be very valuable for developing and creating effective strategies to prevent and manage weight-related stigmatization.

## Conclusion

This is one of the first studies investigating the attitudes of experienced HR professionals towards obese individuals. We found a pronounced stigmatization of obese individuals, especially of women, by HR professionals. This stigmatization has serious socio-economic, psychosocial and health consequences, and therefore, is a priority for public health and obesity care.

## Competing interests

The authors declare that they have no competing interests.

## Authors’ contributions

Conception and design of the study: KEG, ST, AT, SZ. Data acquisition: AT. Data analysis and interpretation: KEG, DW, SZ, CZ, NS, FWH, AT. Manuscript drafting: AT, KEG. Critical revision of the manuscript for important intellectual content: DW, SZ, CZ, NS, FWH, AT, ST. Statistical analysis: KEG, AT, NS. Obtaining funding: SZ, CZ, FWH. Administrative, technical or material support: SZ. Supervision: FWH, SZ, AT. All authors read and approved the final manuscript.

## Pre-publication history

The pre-publication history for this paper can be accessed here:

http://www.biomedcentral.com/1471-2458/12/525/prepub
